# Bugan Rongjin decoction alleviates inflammation and oxidative stress to treat the postmenopausal knee osteoarthritis through Wnt signaling pathway

**DOI:** 10.1186/s12938-021-00939-8

**Published:** 2021-10-13

**Authors:** Sheng Wang, Pei Ding, Xiaopeng Xia, Xuexian Chen, Daguo Mi, Shuijie Sheng, Fulong Gu, Zhongwei Li, Kelei Su, Yuwei Li

**Affiliations:** 1 Department of Orthopedics and Traumatology, Nantong TCM Hospital Affiliated to Nanjing University of Chinese Medicine, Nantong, 226001 Jiangsu China; 2 Department of Pediatrics, Nantong TCM Hospital Affiliated to Nanjing University of Chinese Medicine, Nantong, 226001 Jiangsu China; 3 Department of Science and Education, Nantong TCM Hospital Affiliated to Nanjing University of Chinese Medicine, Nantong, 226001 Jiangsu China; 4 Department of Respiratory Medicine, Affiliated Hospital of Integrated Traditional Chinese and Western Medicine, Nanjing University of Chinese Medicine, Number 100, Cross Street, Hongshan Road, Qixia District, Nanjing, 210028 Jiangsu China; 5 Department of Orthopedics and Traumatology, Suzhou TCM Hospital Affiliated to Nanjing University of Chinese Medicine, Number 18 Yangsu Road, Suzhou, 215000 Jiangsu China

**Keywords:** Bugan Rongjin decoction, Inflammation, Oxidative stress, Wnt signaling pathway, Postmenopausal knee osteoarthritis

## Abstract

**Background:**

Traditional Chinese medicine has been found effective for the therapy of knee osteoarthritis (KOA). This study was aimed at investigating the underlying mechanism of Bugan Rongjin decoction (BGRJ) in treating the postmenopausal KOA.

**Results:**

Ovariectomized rat model of KOA and LPS-induced chondrocytes were successfully constructed for in vivo and in vitro model of postmenopausal KOA. X-ray and hematoxylin–eosin (H&E) staining showed that BGRJ alleviated pathological damage of articular cartilage in OVX rats with KOA. In addition, BGRJ inhibited inflammation and oxidative stress through decreasing the levels of serum IL-6, IL-1β, TNF-α and NO and regulated Wnt signaling pathway by downregulating the expression of Wnt5a and β-catenin and upregulating the expression of Sox9 and Collagen II in cartilage tissue, detected by immunohistochemistry (IHC) and western blot analysis. Furthermore, Wnt5a silencing reduced the apoptosis of LPS-induced ADTC5 cells, which was further suppressed by the combination of downregulation of Wnt5a and BGRJ.

**Conclusions:**

In summary, BGRJ alleviates inflammation and oxidative stress to treat the postmenopausal KOA through Wnt signaling pathway.

## Introduction

Knee osteoarthritis (KOA) is the most common bone degenerative disease in middle-aged and elderly people. It is mainly manifested as knee stiffness, pain, swelling, joint deformity, decreased muscle strength of lower limbs and limited activity [1]. Severe cases can affect the patient's daily self-care ability, which is one of the reasons for the elderly’s disability [2]. With the accelerating process of population aging, the incidence of KOA is also increasing year by year, which greatly increases the economic burden of society and family [3]. In China, 50% ~ 60% of people over 60 years old suffer from KOA [4]. The prevalence of KOA is significantly higher in postmenopausal women than in men of the same age group due to the decrease in estrogen levels in postmenopausal women [5]. The current treatments, such as non-steroidal anti-inflammatory drugs, hyaluronic acid injections, and arthroplasty were demonstrated to be effective, but also accompanied with certain side effects [6]. Furthermore, there is no recommended routine treatment for KOA.

Recently, some promising elements and Chinese herbal compound have been confirmed to slow down the progression of KOA, promote cartilage repair, and alleviate knee joint disorders [7]. KOA belongs to Chinese medicine, “Bi Syndrome”, “Bi-bone” areas, which refers to the pain and stiffness or malfunction of the joints [8]. Wind–cold–damp–hot impediment causes extremities clonus, knee pain and obstruction of flexed and extended, which leaded to a long time existence of Bi syndrome, further damage to the liver and kidney [9]. Zeng et al. [10] analyzed the rule of medication through data mining and concluded that nourishing liver and kidney, and removing blood stasis and freeing vessel was the main treatment for KOA. Liangchun Zhu, a master of Chinese medicine, has considered that the treatment of KOA can be discussed from the deficiency of liver Yin and loss of nutrient of tendons [11].

Bugan Rongjin decoction (BGRJ) is supplemented and improved basing on the Chen Shiduo’s Yangjin Tang. The main ingredients of BGRJ are *Fried Radix Paeoniae Alba, Rehmannia, Radix Ophiopogonis, Fried Zizyphus jujube, Morinda officinalis, Angelica sinensis, Papaya, Achyranthes bidentata Blume, Eupolyphaga sinensis Walker and Lumbricus*. Paeoniflorin (PF), the main substance of glucosides in *Radix Paeoniae Alba*, can alleviate the hepatic ischemia/reperfusion (I/R) injury by suppressing inflammation [12]. Catalpol, an iridoid glycoside isolated from the root of *Rehmannia*, protects against osteoarthritis by inhibiting inflammation, oxidative stress and apoptosis of chondrocytes [13]. Monotropein, an iridoid glycoside isolated from the roots of *Morinda officinalis*, suppresses the apoptosis and inflammation in chondrocytes, which may be useful for the treatment of osteoarthritis [14]. Angelica sinensis polysaccharide has confirmed to exert antioxidant effect on the oxidative stress in human osteoarthritis [15]. Study has evidenced that *Achyranthes bidentata Blume* extracts have anti-osteoarthritis and anti-inflammatory effects [16, 17]. *Eupolyphaga sinensis Walker* is an ingredient of Huoxuezhitong capsule which has proved to alleviate monosodium iodoacetate-induced osteoarthritis [18]. Therefore, BGRJ is used in the treatment of osteoarthritis.

In this study, we aimed at investigating the underlying mechanism underlying BGRJ in treating the postmenopausal KOA. We constructed the ovariectomized rat model of KOA and cultivated lipopolysaccharide (LPS)-induced chondrocytes to explore the therapeutic effects of BGRJ through regulating inflammatory cytokines and Wnt signaling pathway, which may be potential candidates for the therapy and cure of human postmenopausal KOA.

## Results

### BGRJ improves KOA in OVX rats

**Fig. 1:**
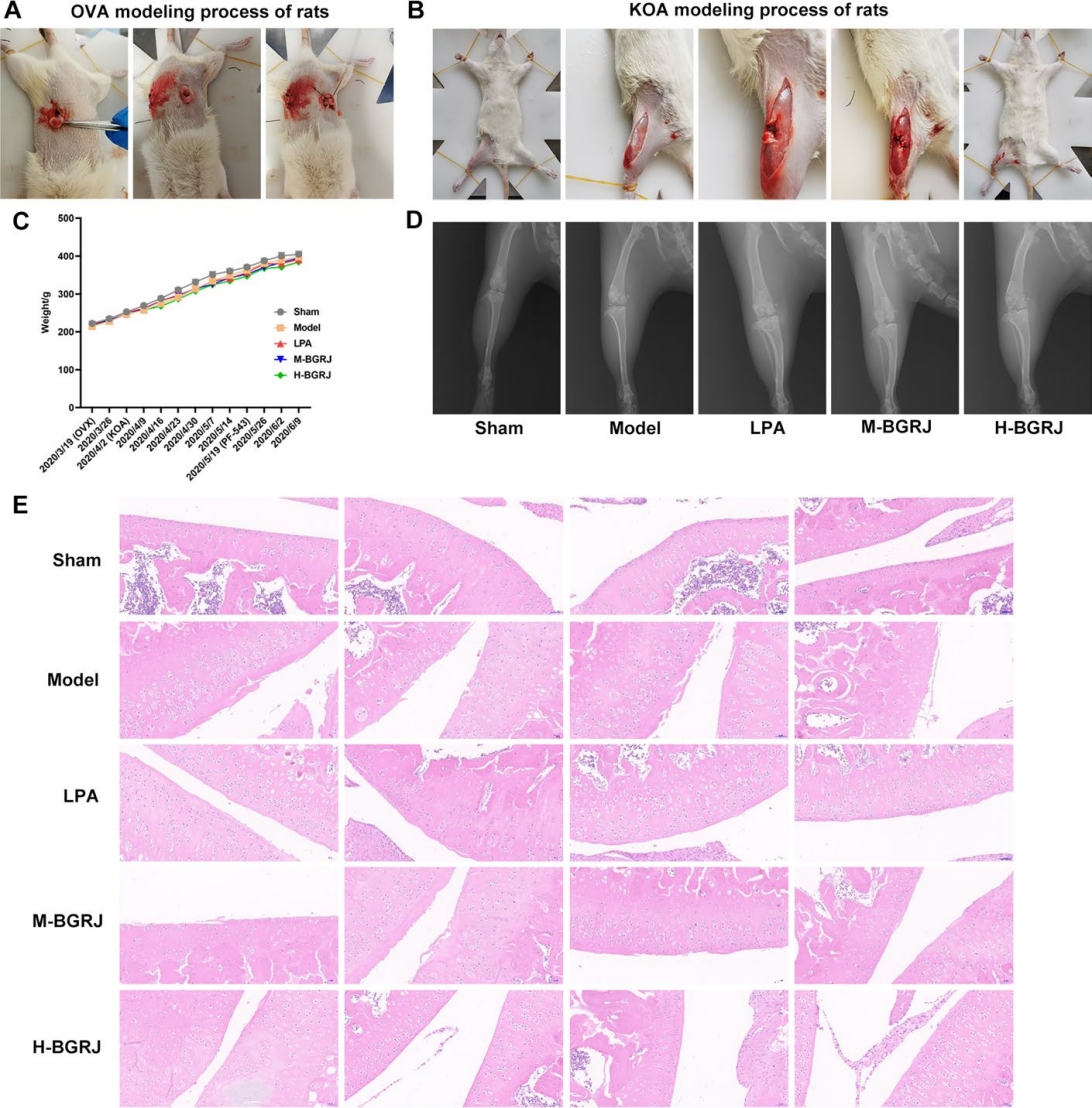
BGRJ improves KOA in OVX rats. **A** OVA modeling process of rats. **B** KOA modeling process of rats. **C** Body weight of rats was recorded after various treatments. **D** The condition of articular cartilage was observed by X-ray. **E** The condition of articular cartilage was observed by H&E

OVA modeling process of rats is shown as Fig. 1A and KOA modeling process of rats was shown as Fig. 1B. During the experiment, the body weight of rats in each group changed steadily, and each treatment had no obvious effect on the body weight of rats (Fig. 1C). The condition of articular cartilage in these groups was observed by X-ray (Fig. 1D), which was consistent with the results of H&E (Fig. 1E). The articular cartilage in the Sham group had normal structure, smooth surface without wear and fissure, normal morphology and number of chondrocytes and regular arrangement. In the model group, there were lesions on the surface of articular cartilage, cracks and faults, severe deletion, local reduction and disordered arrangement of chondrocytes. In the LPA group, the articular cartilage was basically flat and the cells were arranged in regular order, but local unsmoothness was still visible. In the M-BGRJ group, the surface of articular cartilage was uneven, with damage and fissures, disordered cell arrangement and reduced cell number. In the H-BGRJ group, the surface of articular cartilage was basically flat, and some coarser cells could be seen locally. The cells were arranged neatly and the morphology was normal.

### BGRJ alleviates inflammation and oxidative stress in serum of OVX rats of KOA

**Fig. 2:**
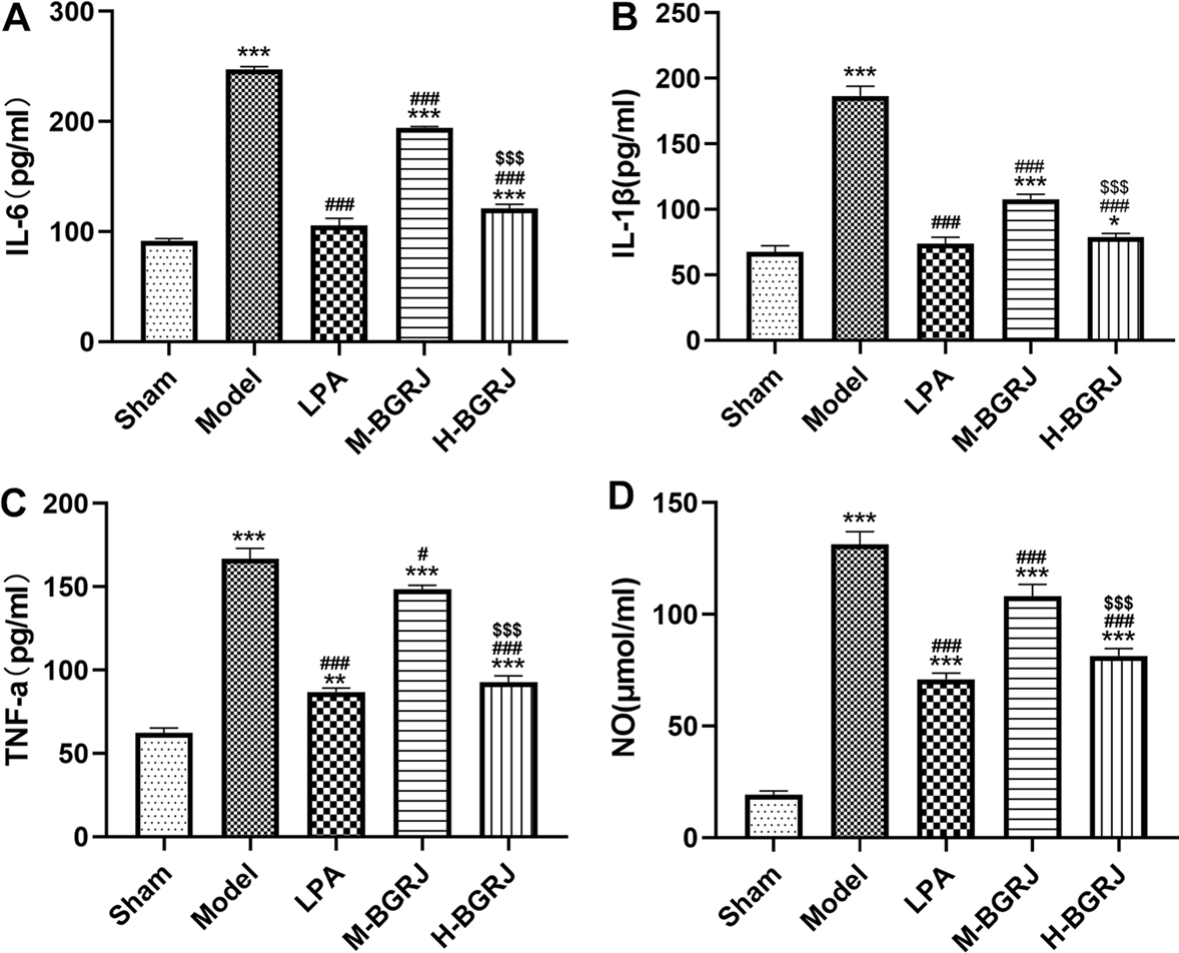
BGRJ alleviates inflammation and oxidative stress in serum of OVX rats of KOA. The levels of IL-6 (**A**), IL-1β (**B**), TNF-α (**C**) and NO (**D**) in rat serum were detected by the commercial kits. *P < 0.05, **P < 0.01 and ***P < 0.001 vs. Sham group. ^#^*P* < 0.05 and ^###^*P* < 0.001 vs. Model group. ^$$$^*P* < 0.001 vs. M-BGRJ group

Compared with the sham group, the expression levels of IL-6, IL-1β and TNF-α in serum were all increased in model group. After treatment of BGRJ, the expression levels of IL-6, IL-1β and TNF-α in serum were decreased (Fig. 2A–C). Similarly, NO level was also obviously enhanced in model group and was down-regulated by BGRJ treatment (Fig. 2D). Moreover, the high-dose BGRJ has a better alleviating effect on the inflammation and oxidative stress.

### BGRJ improves KOA in OVX rats by regulating Wnt signaling pathway

**Fig. 3:**
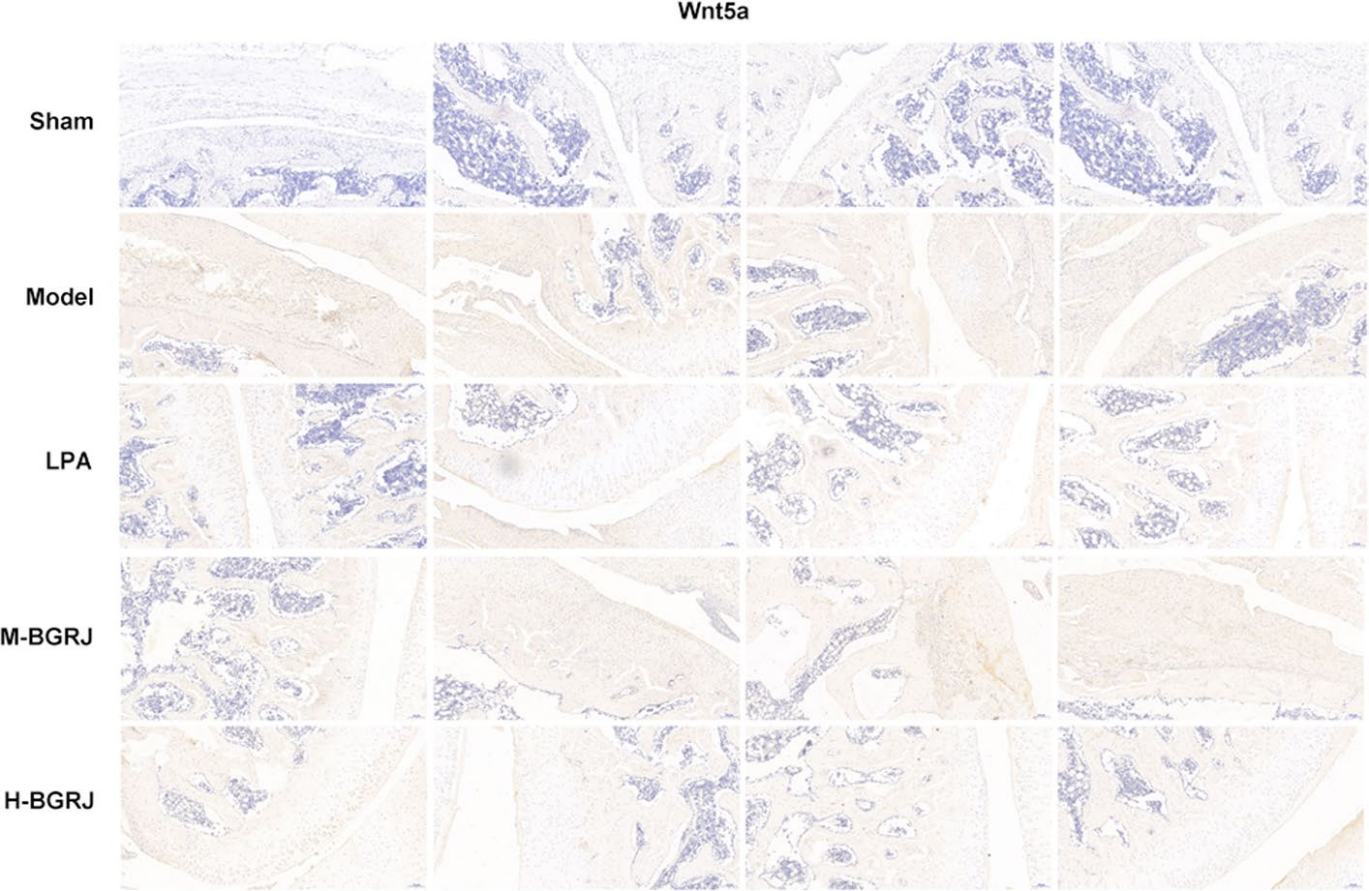
BGRJ improves KOA in OVX rats by regulating Wnt signaling pathway. Wnt5a expression in articular cartilage was detected by immunohistochemistry

**Fig. 4:**
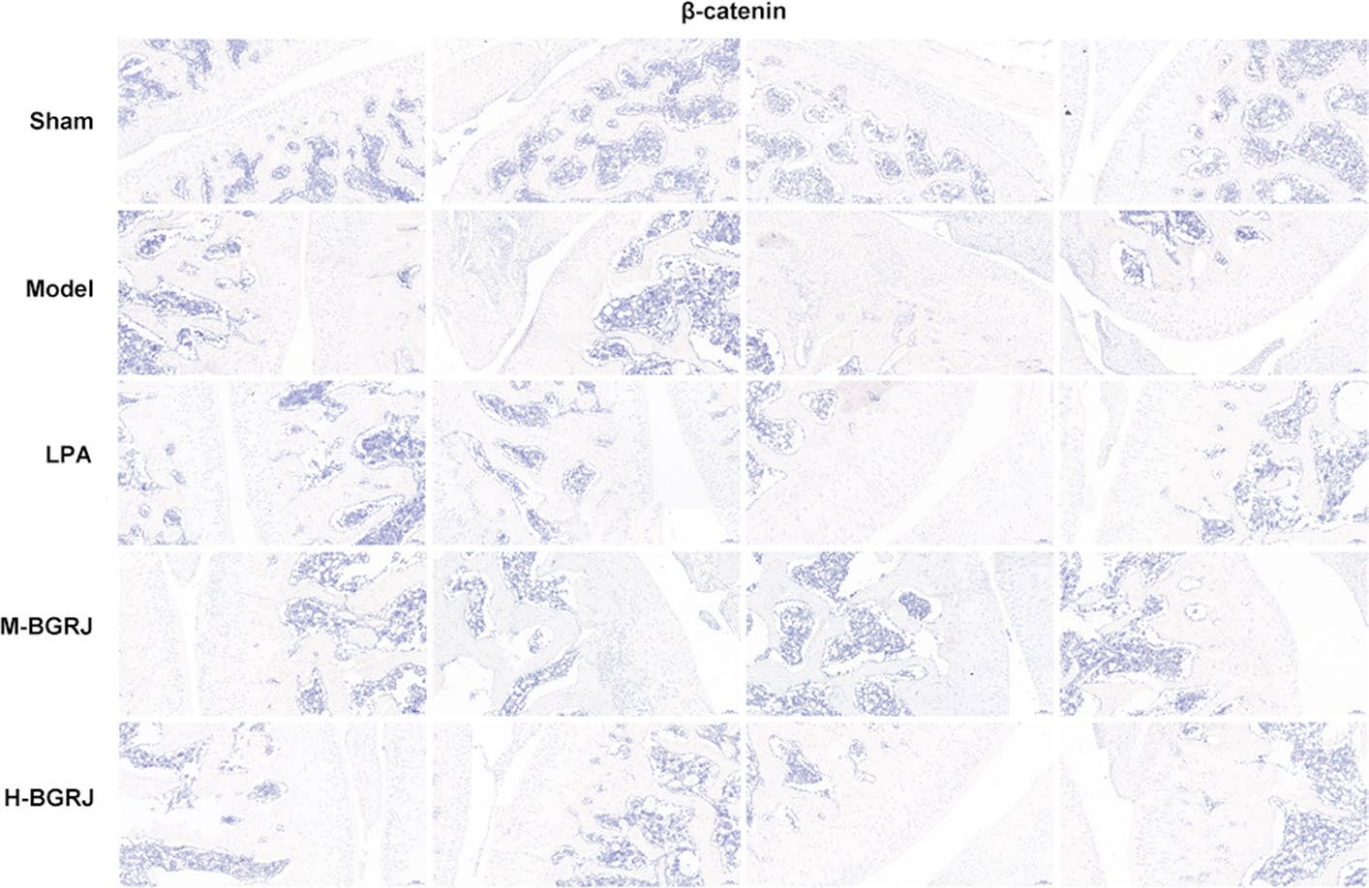
BGRJ improves KOA in OVX rats by regulating Wnt signaling pathway. β-catenin expression in articular cartilage was detected by immunohistochemistry

**Fig. 5:**
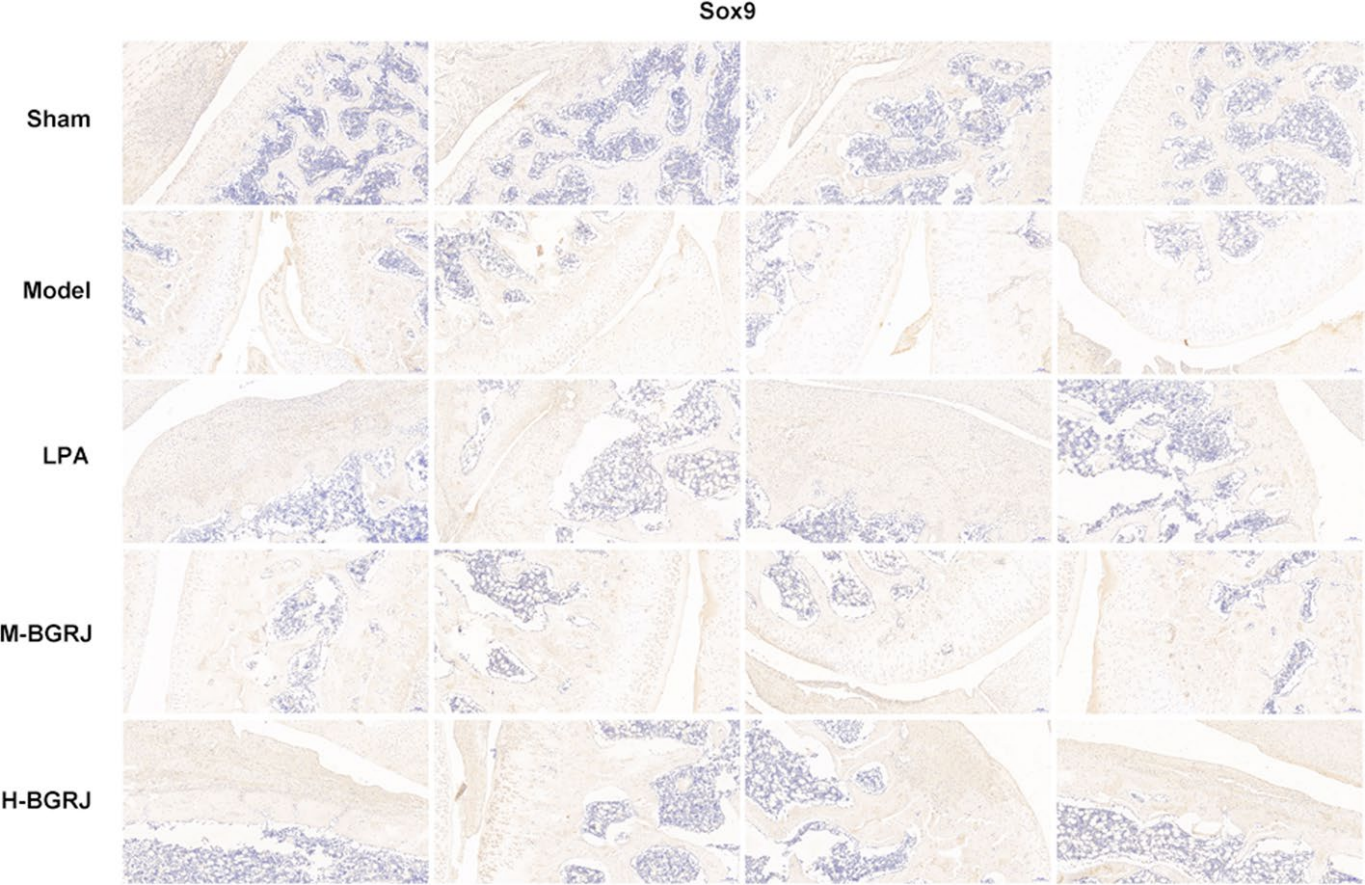
BGRJ improves KOA in OVX rats by regulating Wnt signaling pathway. Sox9 expression in articular cartilage was detected by immunohistochemistry

**Fig. 6:**
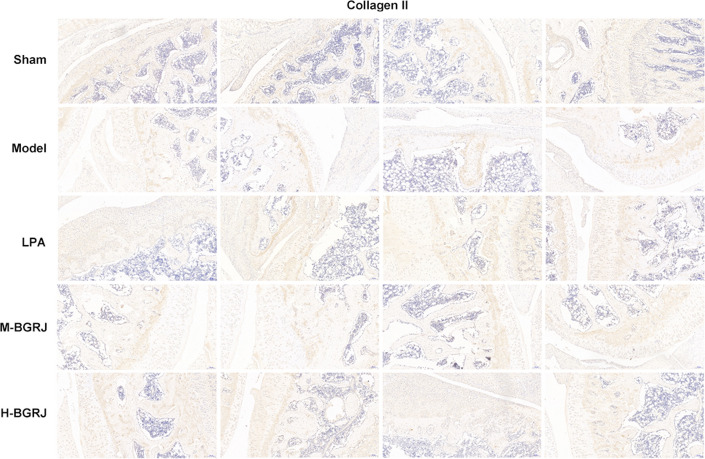
BGRJ improves KOA in OVX rats by regulating Wnt signaling pathway. Collagen II expression in articular cartilage was detected by immunohistochemistry

**Fig. 7:**
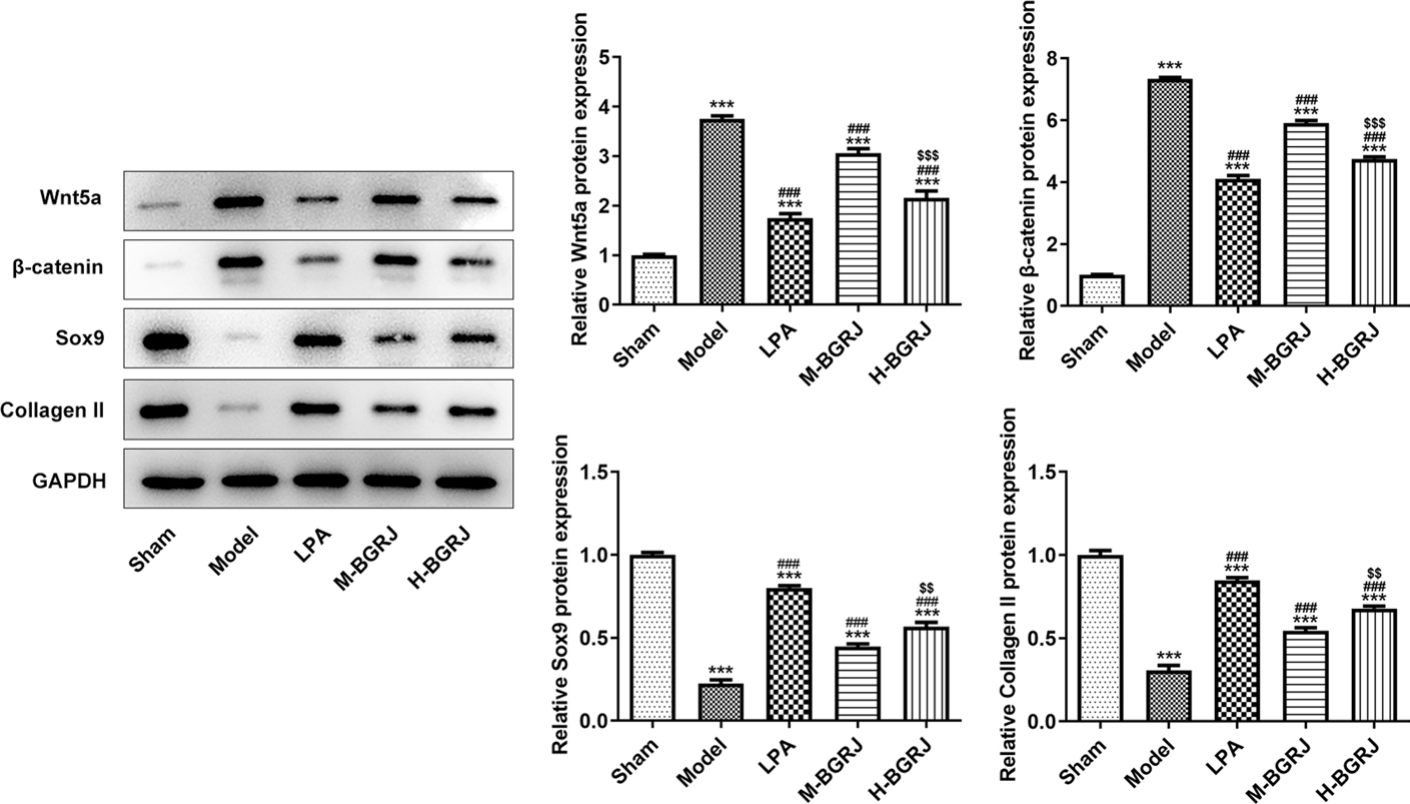
BGRJ improves KOA in OVX rats by regulating Wnt signaling pathway. The expression of Wnt5a, β-catenin, Sox9 and Collagen II in articular cartilage was detected by Western blot analysis. ***P < 0.001 vs. Sham group. ^###^*P* < 0.001 vs. Model group. ^$$^*P* < 0.01 and ^$$$^*P* < 0.001 vs. M-BGRJ group

The expression of Wnt5a (Fig. 3) and β-catenin (Fig. 4) was increased while Sox9 (Fig. 5) and Collagen II (Fig. 6) was decreased in model group. BGRJ partially reversed the changes of Wnt5a, β-catenin, Sox9 and Collagen II in model group. The results of Western blot analysis were also consistent with that of IHC (Fig. 7). Moreover, high-dose BGRJ has better effect than low-dose BGRJ.

### Main components of BGRJ confirmed by HPLC

**Fig. 8:**
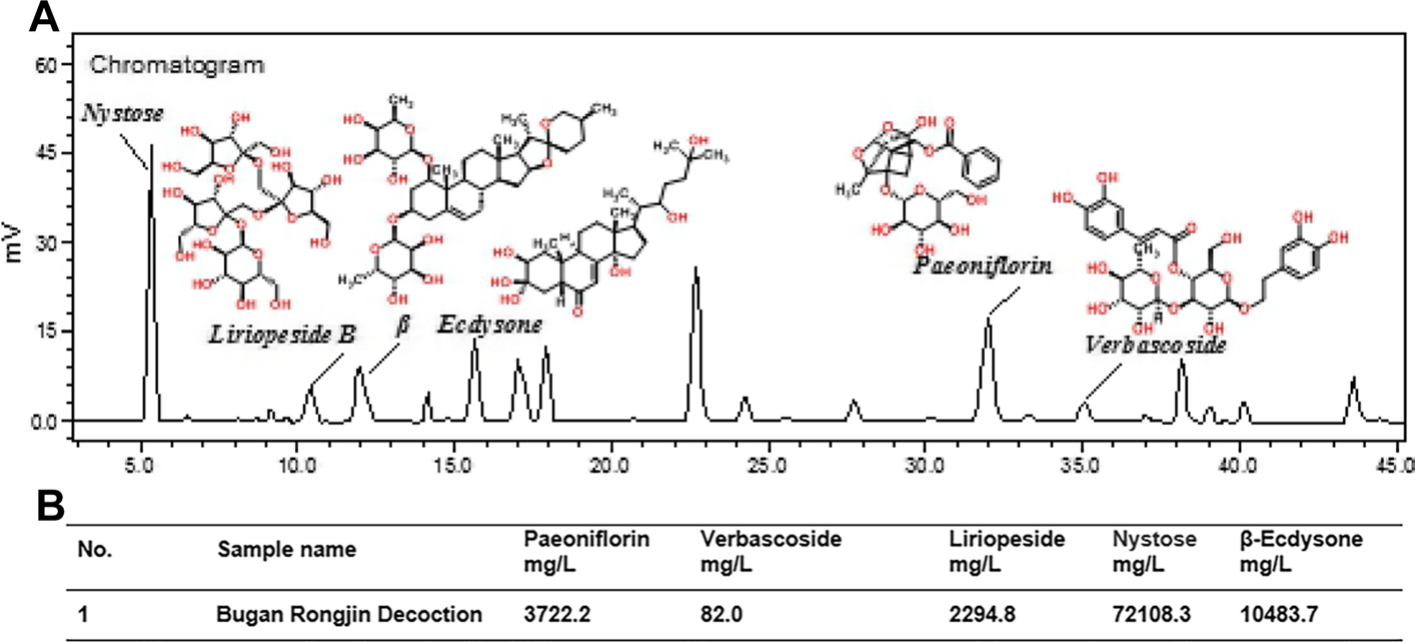
Main components of BGRJ confirmed by HPLC. **A** The main components of BGRJ were confirmed by HPLC. **B** The contents of nystose, liriopeside, β-ecdysone, paeoniflorin and verbascoside in the BGRJ

The main components of BGRJ confirmed by HPLC were nystose, liriopeside, β-ecdysone, paeoniflorin and verbascoside (Fig. 8A). The contents of nystose, liriopeside, β-ecdysone, paeoniflorin and verbascoside in the BGRJ were 72,108.3 mg/L, 2294.8 mg/L, 10,483.7 mg/L, 3722.2 mg/L and 82.0 mg/L (Fig. 8B).

### Wnt5a expression is decreased in ADTC5 cells after transfection

**Fig. 9:**
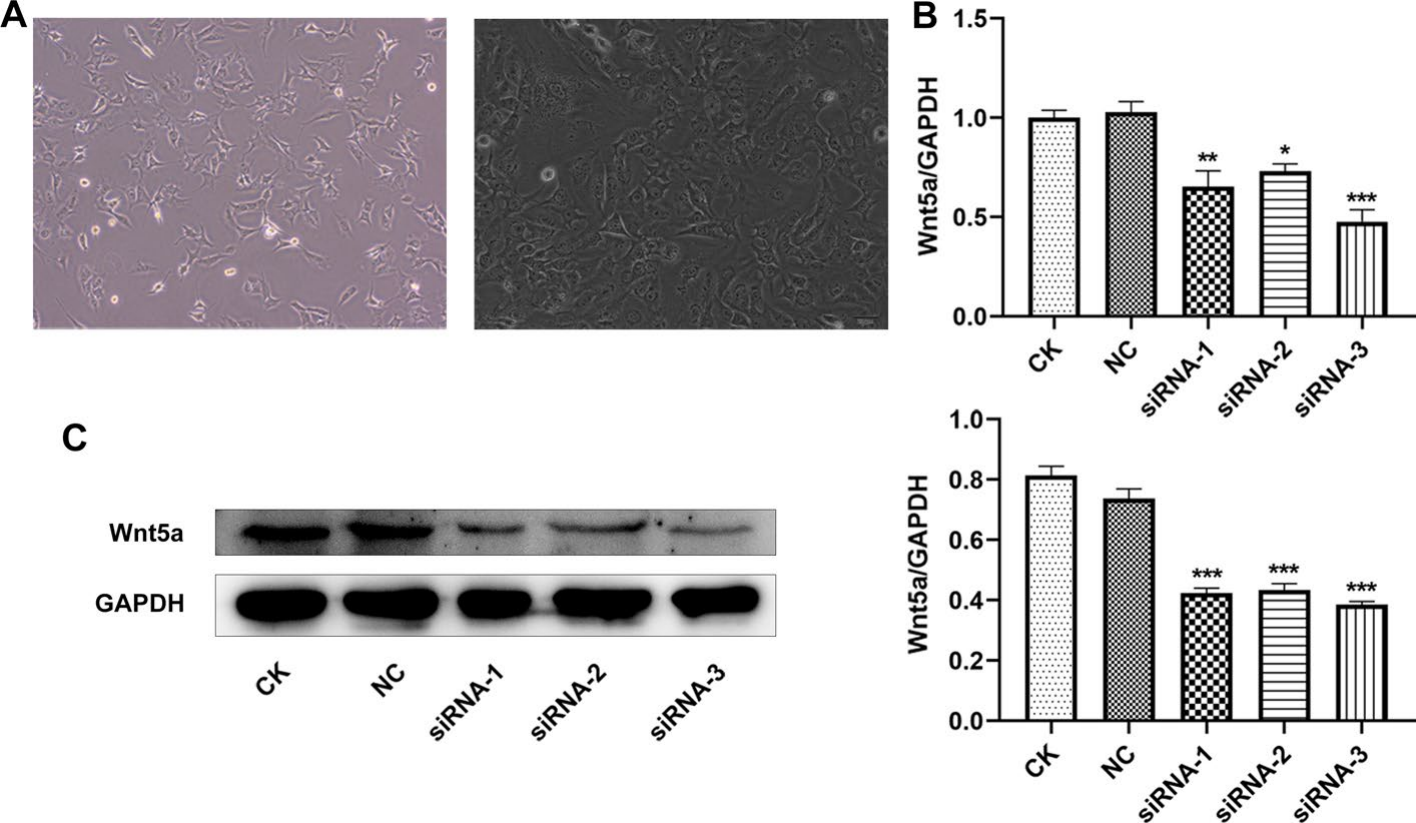
Wnt5a expression is decreased in ADTC5 cells after transfection. **A** The morphology of ADTC5 cells were photographed under microscope. **B** Wnt5a expression in ADTC5 cells after transfection was confirmed by RT-qPCR analysis. **C** Wnt5a expression in ADTC5 cells after transfection was confirmed by Western blot analysis. *P < 0.05, **P < 0.01 and ***P < 0.001 vs. CK group

The morphology of ADTC5 cells was photographed under microscope (Fig. 9A). After ADTC5 cells were transfected with siRNA-NC and wnt5a siRNA, the transfection effects were confirmed by RT-qPCR analysis (Fig. 9B) and Western blot analysis (Fig. 9C). Compared with CK group, the expression level of wnt5a was not significantly affected by siRNA-NC transfection, and was decreased after ADTC5 cells were, respectively, transfected with wnt5a siRNA-1, 2 and 3. We chose wnt5a siRNA-3 for the experiment in the later stage.

### Downregulation of Wnt5a reduces the apoptosis of LPS-induced ADTC5 cells, which is further reduced by BGRJ

**Fig. 10:**
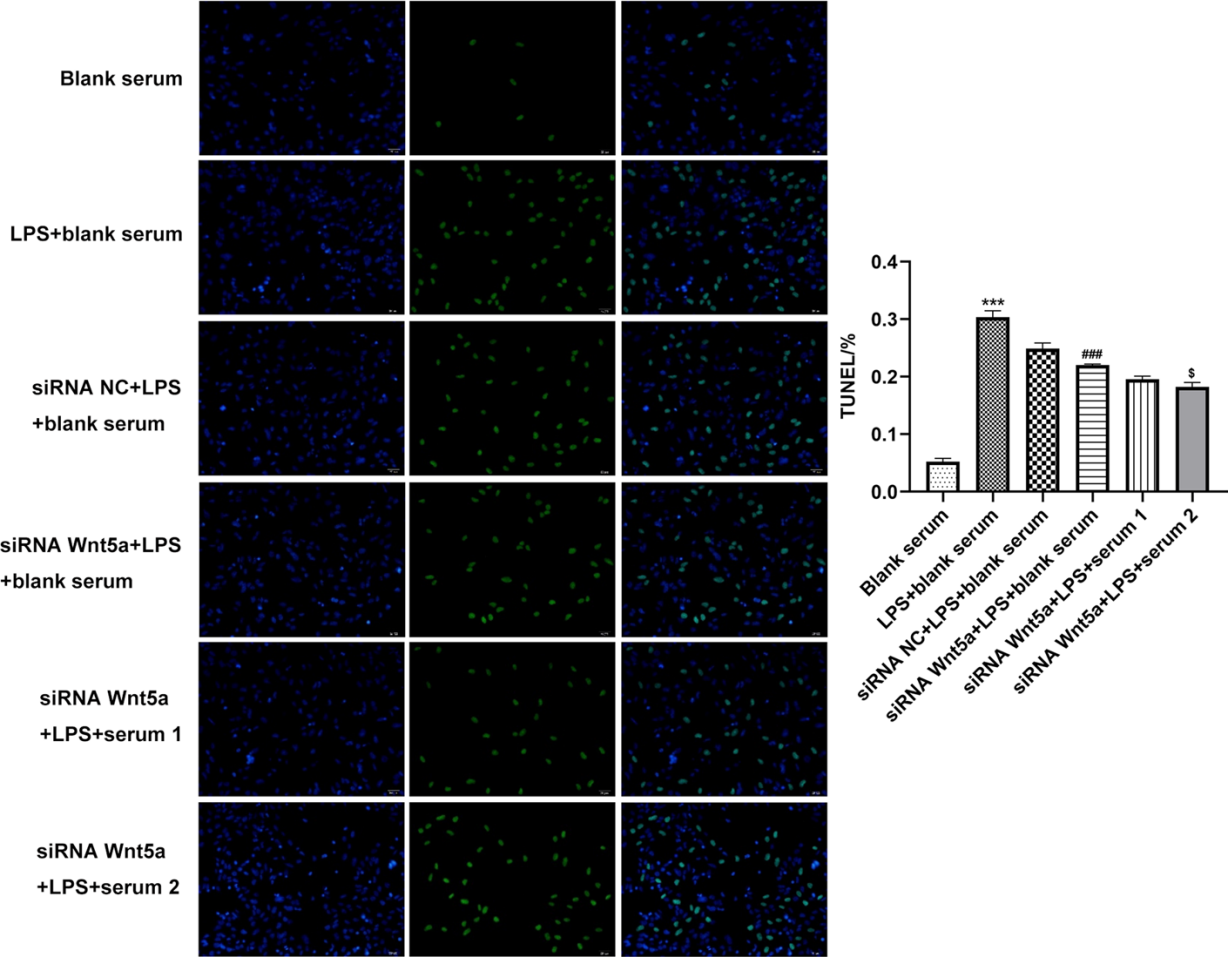
Downregulation of Wnt5a reduces the apoptosis of LPS-induced ADTC5 cells, which is further reduced by BGRJ. The apoptosis of LPS-induced ADTC5 cells was analyzed by TUNEL assay. ***P < 0.001 vs. blank serum group. ^###^*P* < 0.001 vs. LPS + blank serum group. ^$^*P* < 0.05 vs. siRNA-Wnt5a + LPS + blank serum group

LPS induction obviously promoted the apoptosis of ADTC5 cells while downregulation of Wnt5a could suppress the apoptosis of LPS-induced ADTC5 cells. BGRJ could further reduce cell apoptosis, and the high-dose BGRJ could reduce cell apoptosis better (Fig. 10).

### Downregulation of Wnt5a regulates Wnt signaling pathway in LPS-induced ADTC5 cells, which is further regulated by BGRJ

**Fig. 11:**
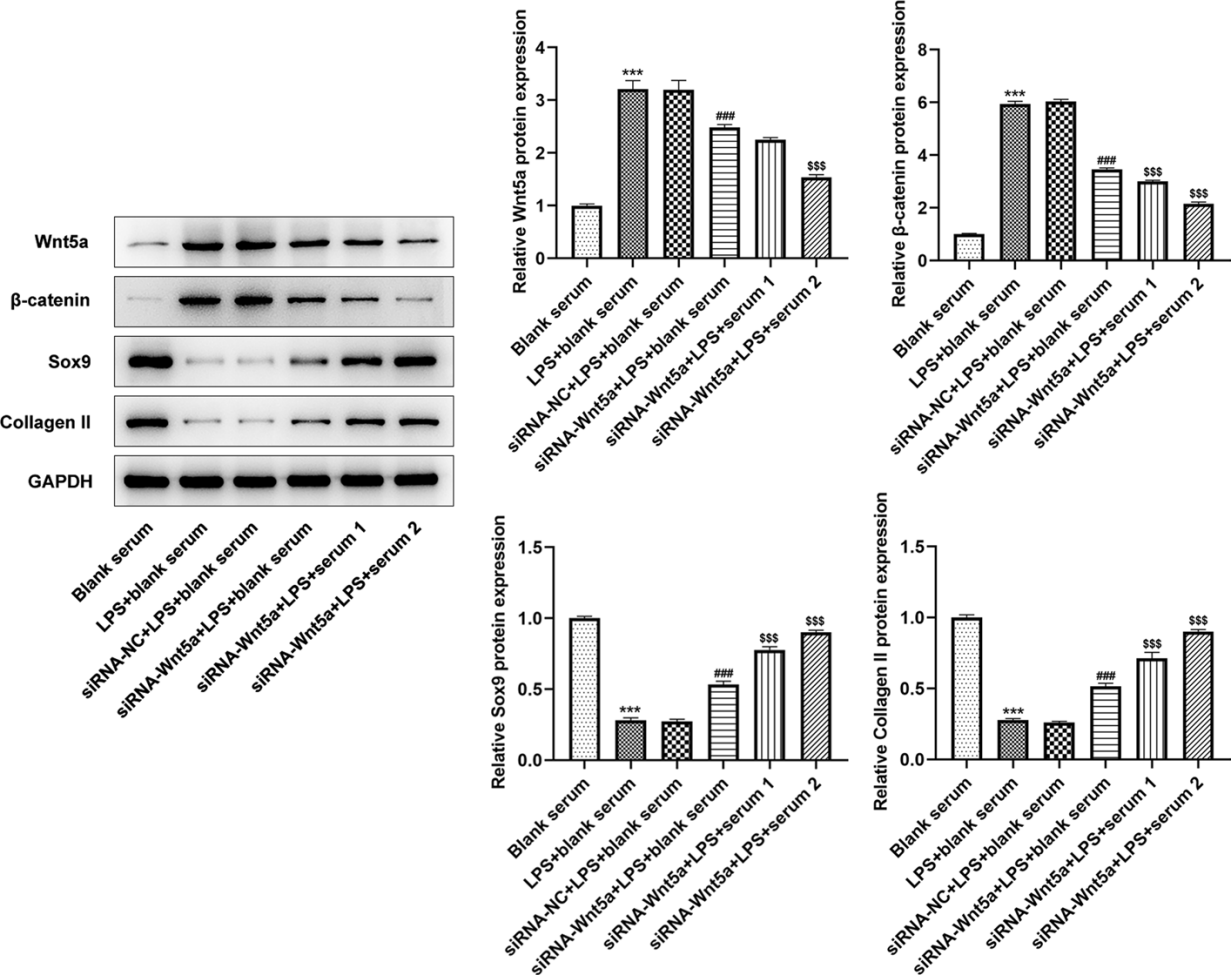
Downregulation of Wnt5a regulates Wnt signaling pathway in LPS-induced ADTC5 cells, which is further regulated by BGRJ. The expression of Wnt5a, β-catenin, Sox9 and Collagen II in LPS-induced ADTC5 cells was detected by Western blot analysis. ***P < 0.001 vs. blank serum group. ^###^*P* < 0.001 vs. LPS + blank serum group. ^$$$^*P* < 0.001 vs. siRNA-Wnt5a + LPS + blank serum group

LPS induction obviously increased the expression of Wnt5a and β-catenin and decreased the expression of Sox9 and Collagen II. Downregulation of Wnt5a inhibited the expression of Wnt5a and β-catenin and promoted the expression of Sox9 and Collagen II, which were further regulated by regulated by BGRJ. High-dose BGRJ had better reversing effect on the expression of Wnt5a, β-catenin, Sox9 and Collagen II in LPS-induced ADTC5 cells (Fig. 11).

## Discussion

Herbal formulae are complex mixtures of many herbs which contain abundant therapeutic compounds. Therefore, the curative effects of herbal formulae are maximized and the toxicity or adverse effects are minimized by interaction of different herbs [19].

Inflammatory reactions are essential in the progression of KOA, which lead to pain, swelling, and dysfunction and derangement of knee joint [20]. Numerous pro-inflammatory cytokines, chemicals and proteins, such as IL-1β, TNF-α, MMPs, hyaluronic acid (HA), SOD, NO, and PGE2, were involved in the occurrence of inflammatory reactions [21, 22]. They not only cause the direct damage to cartilage, but also degrade the extracellular matrix (ECM) and collagen II, which are destroying the knee joint [23]. Herbal therapy significantly regulates the level of many inflammation-related cytokines in the treatment of KOA [24–26]. Here, the levels of IL-6, IL-1β, TNF-α and NO were increased in serum of OVX rats of KOA. BGRJ improved the KOA by inhibiting inflammation.

Wnt/β-catenin signaling pathway has been demonstrated to regulate the bone remodeling independently or interact with other pathways [27]. The canonical Wnt signaling pathway is over-activated in various OA pathogenesis [28]. Under normal conditions, the Wnt/β-catenin signaling pathway is inhibited, and when activated, the overexpression of this pathway promotes the formation of osteoblasts and the release of proteases, during which chondrocytes are degraded, while the block of Wnt signaling induces cartilage formation [29]. The Wnt/β-catenin pathway is very important for the expression of chondrocyte function. β-Catenin and its related molecules play an important role in endochondral ossification and participate in chondrocyte differentiation and proliferation [30]. Herbal therapy can suppress the Wnt/β-catenin signaling pathway, and alleviate dysfunction of knee joint in KOA patients [31]. Its presence indicated that expression of Wnt5a and β-catenin was increased and the expression of Sox9 and Collagen II was decreased in OVX rats of KOA, which was partially reversed by BGRJ. In addition, downregulation of Wnt5a could suppress the apoptosis of LPS-induced ADTC5 cells.

The critical pathological characteristic of KOA is that chondrocytes and cartilage matrix were lost which causes degeneration of articular cartilage. Study demonstrated that herbal therapy could suppress the losses of proteoglycans and collagen II and promote the proliferation of chondrocytes [32]. We have found that the apoptosis of ADTC5 cells was increased after LPS induction, which was inhibited by the treatment of BGRJ for LPS-induced ADTC5 cells, indicating the inhibitory effects of BGRJ on inflammation in KOA development.

There are several limitations in the current study. Firstly, we explore the effects of BGRJ in this study at animal and cell levels but not include the clinical samples, and we will verify our hypothesis through clinical trials in the further study. Moreover, because BGRJ is the mixture and we cannot ensure the most important ingredient that exert the effects in BGRJ. Thus, it is needed to perform the separation and purification to explore the main active constituents in BGRJ. Thirdly, how BGRJ acts on Wnt/β-catenin signaling was not explored in detail in this study and we will investigate the specific mechanism of BGRJ involved in Wnt/β-catenin pathway in next study.

## Conclusion

In conclusion, we demonstrated for the first time that BGRJ alleviates inflammation and oxidative stress in treating the postmenopausal KOA through blocking the Wnt/β-catenin signaling pathway, providing a promising TCM candidate for KOA therapy. The study is expected to develop a new Chinese medicine formula and dosage form mainly composed of BGRJ, which will have great social and economic benefits after clinical application.

## Materials and methods

### Ovariectomized rat model of KOA and BGRJ treatment

Twenty-five 6-month-old female Sprague-Dawley rats were brought from Qing Long Shan animal breeding grounds (Nanjing, China). Rats were raised in standard conditions with a natural light–dark cycle and were normally fed with free access to water. All rats were randomly divided into five groups (*n* = 5): Sham group, Model (ovariectomized rat of KOA) group, LPA (glucosamine potassium sulfate capsules) group, M-BGRJ (medium dosage of BGRJ) group and H-BGRJ (high dosage of BGRJ) group. All the rats except those in sham group were performed bilateral ovariectomy (OVX) to induce an ovariectomized rat model of KOA. After 7 weeks, ovariectomized rat of KOA were, respectively, administered with 3.02 g/kg and 6.04 g/kg BGRJ by gavage in M-BGRJ group and H-BGRJ group, twice each day. In LPA group, ovariectomized rat of KOA were administered with 2.725 mg/kg glucosamine potassium sulfate capsules solution by gavage, twice each day. Rats in sham group and model group were given 0.9% normal saline in the same volume. During the whole experiment, weights of rats were recorded every 7 days. Three weeks after administration, the rats were intraperitoneally anesthetized with 10% chloral hydrate solution. The condition of articular cartilage was observed by X-ray. Blood was collected from abdominal aorta, centrifuged at 3000 r for 15 min, and the supernatant was stored in − 80 °C refrigerator. The distal femur, proximal tibia and medial gastrocnemius of the right lower limb were harvested for further analysis. All experiments were approved by the Animal Care and Use Committee of Nantong TCM Hospital.

### Hematoxylin–eosin (H&E) staining and immunohistochemistry (IHC)

The articular cartilage was fixed in 10% formalin solution for 24 h, decalcified in 15% EDTA-2Na solution at room temperature for 7 days and embedded in paraffin wax. For histological examination, 6-μm sections were deparaffinized and rehydrated using graded ethanol (100%, 90%, and 70%) series and stained with hematoxylin and eosin. For immunohistochemical study, 6 μm sections were treated with 10% H_2_O_2_ for 10 min at 37 °C, followed by incubation of goat serum for 15 min at 37 °C. Then, sections were incubated overnight at 4 °C with the primary antibodies containing Wnt5a, β-catenin, Sox9 and Collagen II. After PBS washing for three times, sections were incubated with goat anti-rabbit secondary antibody for 30 min at 37 °C and incubated with HRP-labeled streptavidin for 15 min at 37 °C. After washing in PBS for three times, sections were stained with 3, 3 diaminobenzidinetetrahydrochloride (DAB) and nuclei were counterstained with hematoxylin, which were observed under a light microscope.

### ELISA assay and nitric oxide (NO) detection

Serum interleukin-6 (IL-6), interleukin 1-β (IL-1β) and tumor necrosis factor-alpha (TNF-α) levels were measured using the corresponding rat enzyme-linked immunosorbent assay (ELISA) kits (Beyotime) according to the manufacturer’s instructions. Serum NO was determined using NO assay kit (Beyotime).

### Preparation of medicine serum

Eighteen adult Sprague–Dawley female rats were randomly divided into three groups (*n *= 6): medicine serum 1 group, Medicine serum 2 group and Drug-free (“blank”) serum group. Rats in medicine serum 1 and 2 groups were, respectively, administered with 1.51 g/kg and 6.04 g/kg BGRJ (0.805 g/mL) by gavage for consecutive 3 days. On the morning of the 4th day, 10 mL of blood was collected from the abdominal aorta 1 h after gavage (fasting and free drinking water 12 h before blood collection) and centrifuged at 2000 r/min. After centrifugation for 10 min, plasma was collected and stored in the − 20 °C refrigerator for later use.

### Cell culture and cell transfection

ATDC5 mouse chondroprogenitor cells were purchased from Sigma Aldrich. ATDC5 cells were cultured in Dulbecco's modified Eagle medium (DMEM) and Nutrient F-12 Ham (1:1) containing 5% fetal bovine serum and 1% antibiotic–antimycotics at 37 °C in an atmosphere of 5% CO_2_. ATDC5 cells were transfected with siRNA-NC, wnt5a siRNA-1, wnt5a siRNA-2 and wnt5a siRNA-3 using Lipofectamine^®^ RNAi MAX Reagent. After transfection for 48 h, the transfection effects of cells in each group were detected by RT-qPCR. ATDC5 cells in control check (CK) group were normally cultured.

### RT-qPCR analysis

Total RNA in ATDC5 cells was isolated using the RNA easy Mini kit and reverse transcription was performed using the Quanti-Tec^®^ reverse transcription kit for first-strand cDNA synthesis. Then, cDNAs were amplified by PCR assays with SYBR Green Mix kits, and the relative wnt5a expression was standardized with 2^−ΔΔCt^ method. The PCR program was 95 °C for 10 min, followed by 44 cycles of denaturation at 95 °C for 10 s, annealing at 60 °C for 20 s and extension at 72 °C for 10 s. The primers employed were as follows: wnt5a forward, 5′-TTCAACTCCCCGACCACG-3′ and reverse, 5′-ATAGCCACGCCCACAGCA-3′; GAPDH forward, 5′-GGTGAAGGTCGGTGTGAACG-3′ and reverse 5′-CTCGCTCCTGGAAGATGGTG-3′.

### TUNEL assay

When ATDC5 cells reached the logarithmic growth stage, the cell concentration was adjusted to 1 × 10^5^ cells/mL, and the cells were seeded into 48-well plate with 0.3 mL per well. ATDC5 cells were transfected with siRNA-NC and wnt5a siRNA as above described. siRNA-NC transfected ATDC5 cells were induced by LPS (5 μg/mL) and treated with blank serum in siRNA-NC + LPS + blank serum group. wnt5a siRNA transfected ATDC5 cells were induced by LPS (5 μg/mL) and, respectively, treated with blank serum (wnt5a siRNA + LPS + blank serum group), medicine serum 1 (wnt5a siRNA + LPS + medicine serum 1 group) and medicine serum 2 (wnt5a siRNA + LPS + medicine serum 2 group). ATDC5 cells were only treated with blank serum in blank serum group and ATDC5 cells were induced by LPS (5 μg/mL) and treated with blank serum in LPS + blank serum group. After 24-h treatment, TUNEL assay was done on all ATDC5 cells. ATDC5 cells were washed with PBS once and fixed with 4% paraformaldehyde for 30 min. After PBS washing, ATDC5 cells were incubated in PBS containing 0.3% Triton X-100 at room temperature for 5 min. 50 μL TUNEL working solution was added to each well for incubation at 37 °C for 1 h in the dark. After washing with PBS for three times, ATDC5 cells were incubated in DAPI at 37 °C for 10 min in the dark. Apoptotic cells (green fluorescence staining) were detected by fluorescence microscope.

### Western blot analysis

Total proteins in ATDC5 cells and cartilage tissues were extracted in RIPA lysis buffer and the lysis extract was centrifuged at 12,000 r/min for 5 min at 4 °C. The concentration of total protein supernatant was determined using BCA assay kit. 25 μg protein samples were separated with 12% SDS-PAGE and transferred to a polyvinylidene-difluoride (PVDF) membrane using a wet electroblotting apparatus. Blocked with 5% skimmed milk powder for 1 h at room temperature, membranes were probed overnight at 4 °C with primary antibodies against Wnt5a, β-catenin, Sox9, Collagen II and GAPDH. Next day, membranes were incubated with horseradish peroxidase-linked IgG secondary antibody at 1:5000 dilution for 2 h at room temperature. Band intensities were obtained using a digital image scanner.

### Statistical analysis

SPSS version 22.0 was used for the analysis of data which were presented as mean ± standard deviation (SD). The significance of differences between multiple groups was evaluated by one-way analysis of variance (ANOVA) followed by post hoc Tukey’s test. *P* < 0.05 was considered statistically significant.

## Data Availability

The experimental data will be available on the request.

## Abbreviations

KOA: Knee osteoarthritis
BGRJ: Bugan Rongjin decoction
IL-6: Interleukin-6
TNF-α: Tumor necrosis factor-alpha
NO: Nitric oxide
H&E: Hematoxylin–eosin
IHC: Immunohistochemistry
PF: Paeoniflorin
I/R: Ischemia/reperfusion
LPS: Lipopolysaccharide
HA: Hyaluronic acid
ECM: Extracellular matrix
OVX: Ovariectomy
ELISA: Enzyme-linked immunosorbent assay
DMEM: Dulbecco’s modified Eagle medium
CK: Control check
PVDF: Polyvinylidene difluoride
SD: Standard deviation
ANOVA: Analysis of variance
